# Visceral adipose tissue during pregnancy in women with overweight or obesity and offspring metabolic health

**DOI:** 10.1038/s41366-025-01872-9

**Published:** 2025-08-25

**Authors:** Christina Sonne Mogensen, Faidon Magkos, Elizaveta Chabanova, Christian Mølgaard, Nina Rica Wium Geiker

**Affiliations:** 1 Department of Nutrition, Exercise and Sports, Faculty of Science, University of Copenhagen, Copenhagen, Denmark; 2 Department of Radiology, Copenhagen University Hospital Herlev-Gentofte, Herlev, Denmark; 3 Centre for Childhood Health, Copenhagen, Denmark; 4 Dietetic and Clinical Nutrition Research Unit, Copenhagen University Hospital Herlev-Gentofte, Herlev, Denmark

**Keywords:** Physiology, Medical research, Risk factors, Paediatrics, Weight management

## Abstract

**Background:**

Pre-pregnancy obesity is linked to an increased risk of adverse maternal and neonatal outcomes, and an increased likelihood of offspring obesity later in life. Accumulation of visceral adipose tissue (VAT) has been reported to be more detrimental to health outcomes than generalized obesity. Therefore, we investigated the association between maternal VAT and the metabolic health of offspring at birth.

**Methods:**

This study was a secondary analysis of a dietary randomized controlled trial. Magnetic resonance imaging was performed in gestational weeks (GW) 15, 32, and at birth in a cohort of 119 pregnant women with a pre-pregnancy body mass index ranging from 28–45 kg/m^2^. Offspring anthropometric measurements and cord blood samples were collected at birth. Linear regression models were applied to evaluate the association between maternal VAT and offspring outcomes. Analysis of covariance was utilized to compare offspring outcomes among mothers who exhibited either an increase or a decrease in VAT volume from GW 15 to birth.

**Results:**

Absolute maternal VAT during pregnancy was not associated with birthweight; however, women who experienced a reduction in VAT volume during pregnancy entered pregnancy with a higher VAT volume and gave birth to heavier infants by 161 grams (95% CI, 15; 307, *P* = 0.031) compared to those who experienced an increase in VAT during pregnancy. Maternal VAT in GW 15 and at birth was associated with increased insulin concentrations in the offspring by 0.25 pmol/L (95% CI, 0.03; 0.46, *P* = 0.026) and 0.23 pmol/L (95% CI, 0.02; 0.44, *P* = 0.035), respectively, per 1 cm^3^ increase in VAT.

**Conclusion:**

Maternal VAT, and particularly its change during pregnancy, may influence the fetal metabolic environment, impacting insulin availability and potentially fetal growth in healthy women with overweight or obesity.

## Introduction

Maternal obesity before pregnancy is associated with an increased risk of maternal and neonatal adverse outcomes [1], and is also associated with offspring obesity later in life [2]. Pre-pregnancy body mass index (BMI) is currently used for risk assessment of maternal and offspring complications [3], however, BMI cannot distinguish between muscle and fat tissues and cannot inform on regional fat distribution. Visceral adipose tissue (VAT) is positively associated with BMI but varies largely for the same degree of obesity [4]. Moreover, there is much evidence that accumulation of VAT and ectopic fat is associated with metabolic complications of obesity to a greater extent than indices of whole-body adiposity such as BMI or total body fat [5, 6].

Research has shown that pregnant women experience a threefold greater increase in VAT (measured by dual-energy x-ray absorptiometry) from preconception to postpartum compared to non-pregnant women over a similar timeframe [7]. We recently observed dynamic changes in VAT and subcutaneous adipose tissue (SAT)—measured by magnetic resonance imaging (MRI)— during pregnancy, with VAT exhibiting a U-shaped pattern, decreasing from early to mid/late pregnancy and then rebounding around birth in healthy women with overweight or obesity [8]. Further, it has been reported that VAT increases as the pregnancy progresses among women in different BMI categories. However, the relative changes in VAT and SAT from early to late pregnancy varied by BMI strata, with a larger percentage increase in VAT among women with normal weight [9].

Obesity, and particularly intra-abdominal obesity, leads to greater availability of fatty acids in the maternal circulation, which may be transported to the fetus via the placenta, resulting in increased offspring birthweight [10]. In pregnant women, VAT is associated with an increased risk of pregnancy complications [11], and maternal “central” adipose tissue is positively associated with increased birthweight and increased risk of large for gestational-age infants [10–12]. However, the time-point of assessment during pregnancy varies across studies, and the definition of central adipose tissue is inconsistent, with different techniques having been used, including waist-to-hip ratio and ultrasound. Ultrasound is the most commonly used technique for measuring VAT during pregnancy, however, ultrasound cannot be used in late pregnancy due to the enlarged uterus and fetus. Moreover, ultrasound during pregnancy has not been validated against MRI, the gold standard for measuring abdominal fat [13]. In fact, information about maternal VAT and its relation to offspring weight and metabolic health is scarce. Therefore, we aimed to investigate the association between maternal VAT volume measured by MRI at different time-points during pregnancy with offspring birthweight and metabolic biomarkers in cord blood.

## Methods

### Study design

This study is a secondary analysis of the APPROACH (an optimized programming of healthy children) dietary randomized controlled trial (RCT), conducted from January 2013 to December 2017 at the Copenhagen University Hospital Herlev-Gentofte [14]. The participants received written and oral information about the study before signing an informed consent. The women were randomized to either a high-protein, low-glycemic index diet or a moderate-protein, moderate-glycemic index diet. The MRI examinations were performed in gestational week (GW) 15, 32, and around birth. Data on offspring anthropometry and umbilical cord blood samples were collected at birth. All study procedures were performed in accordance with the Helsinki II Declaration. The study was approved by the Ethical Committee of the Capital Region of Denmark (H-3-2013-119) and was registered at ClinicalTrials.gov (NCT01894139).

### Participants

Pregnant women were included in the APPROACH RCT if they had a planned birth at the Copenhagen University Hospital Herlev-Gentofte, were older than 18 years, had a singleton pregnancy, and had a pre-pregnancy BMI between 28 and 45 kg/m^2^. Women were excluded if they consumed more than 14 units/week of alcoholic drinks, had drug abuse, or had any severe diseases at screening (e.g., diabetes, kidney disease, cancer, liver disease) or a disease developed during pregnancy evaluated to impact the outcomes, e.g. gestational diabetes mellitus. A total of 279 were eligible to participate in the RCT; however, women were included in this analysis if they completed the MRI examinations at all three time-points, leaving 119 women eligible for data analysis. Out of the 208 women who gave birth to a living infant, the intervention group consumed a diet during their pregnancy with approximately 25% of energy as protein, and a glycemic index of 45. In comparison, protein intake in the control diet group was 18%, and the glycemic index was 54 [15].

### Exposure

MRI measured VAT in GW 15 (−173 ± 51 days), GW 32 (−54 ± 37.5 days), and at birth (8 ± 36.5 days). The days were calculated as the intervals between the MRI and the birthdate. The MRI examinations were conducted using an open 1T Panorama HFO MR-imaging system (Philips Medical Systems, Best, the Netherlands) and performed with a Sense Body Large or XLarge coil. A transverse slice with a thickness of 10 mm was acquired at the midpoint of the third lumbar vertebra (L3). The volume of VAT at L3 was measured in cubic centimeters (cm^3^) using a segmentation tool on the Philips ViewForum workstation.

### Outcomes

Birthweight was recorded immediately after birth by midwives at Copenhagen University Hospital Herlev-Gentofte, as part of standard hospital procedures. Birthweight was measured to the nearest 10 grams using a medical beam scale (Tanita, Illinois, USA). BMI z-score was calculated according to the World Health Organization (WHO) standards for sex-specific BMI [16].

Umbilical cord blood samples were collected and stored at −70 °C. Once all samples were collected, plasma was analyzed for glucose and triglycerides, and serum was analyzed for insulin and C-peptide. Glucose and triglyceride levels were measured by colorimetry on a Pentra 400 analyzer (Horiba ABX SAS, France). Insulin and C-peptide levels were determined using an Immulite 2000 system (Siemens Healthcare, UK). For insulin concentrations <14.4 pmol/L (the limit of detection), serum samples were reanalyzed with an ultrahigh-sensitive insulin ELISA kit from Mercodia AB (cat. no. 10-1132-01).

### Covariates

Maternal age and height were obtained at the screening visit. Height was measured in duplicate by a wall-mounted stadiometer (Seca, Germany) to the nearest 0.5 cm. Pre-pregnancy weight was recorded by the women’s general physician at the beginning of pregnancy or self-reported by the participant. Pre-pregnancy BMI was calculated as pre-pregnancy weight (kg) divided by the square of the height (m^2^). Pre-pregnancy weight correlated with measured weight at GW 15 (R^2^ = 0.95).

Maternal weight was measured to the nearest 0.1 kg by a medical scale (Tanita, Illinois, USA) nine times from GW 15 to the end of pregnancy. Gestational weight gain (GWG) in GW 15 was determined by subtracting pre-pregnancy weight from the weight recorded at GW 15. Likewise, GWG in GW 32 was calculated by subtracting pre-pregnancy weight from the weight in GW 32. Total GWG was calculated as the last measured weight during pregnancy minus the pre-pregnancy weight. Parity information was collected via questionnaires.

### Statistics

A complete case analysis was utilized for women who performed all three MRI scans in GW 15, 32, and at birth. The Shapiro-Wilk test was used to determine whether the data were normally distributed. Maternal and offspring characteristics were presented as mean standard deviation (SD) for normally distributed data, median (Q1; Q3) for skewed distributions, or absolute numbers and percentages (%) for categorical variables. To examine the associations of maternal VAT in GW 15, 32, and at birth with offspring outcomes, we utilized an unadjusted linear regression model (model 1) and a fully adjusted multilinear regression model (model 2, adjusted for randomization group, gestational age, parity, pre-pregnancy BMI, and GWG). One infant with a glucose level <1 mmol/L was excluded from the analysis as this level was unrealistically low. The residuals in the linear regression models were visually checked for normal distribution. Women who experienced either a reduction or an increase in VAT volume during pregnancy (calculated as VAT volume at birth minus VAT volume in GW 15) were categorized into two groups. Analysis of covariance (ANCOVA) was used to investigate differences in offspring outcomes between these groups adjusted for parity, maternal age, pre-pregnancy BMI, and GWG. Outcomes in each group were presented as estimated means ± standard error (SE), and the differences in outcomes were calculated as the estimated mean outcome for women who increased their VAT volume minus the estimated mean outcome for women who decreased their VAT volume. A *p* value of 0.05 was used. All statistical analyses were conducted using R (version 4.2.2).

## Results

A complete case analysis was conducted on the 119 women who completed all three MRI scans in GW 15, 32, and at birth. The participating women had a mean (SD) age of 30.8 (4.54) years, a mean (SD) pre-pregnancy BMI of 33.9 (4.0) kg/m², and a median (Q1; Q3) GWG of 6.8 (3.1;10.5) kg (Table 1). There were no significant differences in maternal age, pre-pregnancy BMI, or GWG between those who completed all MR scans (*n* = 119) and those who did not (*n* = 89). During mid- and late pregnancy, the average maternal energy intake was 7582 kJ/day, with macronutrient contributions of 46.4% from carbohydrates, 31.7% from fat, and 21.9% from protein (Supplementary Table S1).

**Table 1:** Maternal and offspring characteristics.

Maternal	*N*	
Maternal age (years)	119	30.8 (4.54)
Parity, nulliparous, *n* (%)	119	60 (50.9)
Pre-pregnancy BMI (kg/m^2^)	119	33.9 (4.0)
Randomization group, HPLGI, *n* (%)	119	66 (55.5)
GWG in GW 15 (kg)	117	0.47 (2.88)
GWG in GW 32 (kg)	117	4.42 (4.94)
GWG, total (kg)	119	6.8 (3.1; 10.5)
VAT GW 15 (cm^3^)	119	123 (95; 156)
VAT GW 32 (cm^3^)	119	82 (62.5; 106.5)
VAT at birth (cm^3^)	119	108 (82.5; 138.5)

Offspring	*N*	
Gestational age	119	283 (277; 288)
Birthweight (g)	118	3616 (448)
BMI z-score	118	−0.08 (0.85)
Glucose (mmol/L)	77	5.56 (1.30)
Insulin (pmol/L)	76	32.7 (20.4; 64.7)
C-peptide (pmol/L)	77	324 (223; 467)
Triglycerides (mmol/L)	78	0.52 (0.41; 0.61)

Data were presented as mean (SD) for normally distributed variables, as median (Q1; Q3) for skewed variables, or as absolute numbers and percentages (%) for categorical variables.

*GW* gestational week, *GWG* gestational weight gain, *HPLGI* high-protein, low-glycemic index, *VAT* visceral adipose tissue.

Maternal VAT measured in GW 15, 32, and at birth showed no significant association with offspring birthweight. However, a positive association was observed between maternal VAT in GW 32 and offspring BMI z-score. Maternal VAT measured in GW 15 and at birth was associated with increased offspring insulin at birth, but no associations were observed with C-peptide, glucose, or triglyceride concentrations after adjusting for confounding variables (Table 2).

**Table 2:** Maternal visceral adipose tissue during pregnancy and offspring outcomes.

		Model 1		Model 2	
Outcome	Gestational week	Estimate (95% CI)	*P* value	Estimate (95% CI)	*P* value
Birthweight (g)	15	1.41 (−0.37; 3.18)	0.119	1.59 (−0.14; 3.32)	0.072
	32	0.67 (− 1.09; 2.42)	0.453	1.34 (−0.21; 2.88)	0.090
	At birth	−0.12 (−1.90; 1.67)	0.899	0.47 (−1.14; 2.09)	0.560
BMI z-score	15	0.002 (−0.002; 0.005)	0.278	0.003 (−0.00; 0.01)	0.100
	32	0.002 (−0.001; 0.005)	0.224	0.004 (0.00; 0.01)	**0.031**
	At birth	0.001 (−0.002; 0.005)	0.414	0.003 (−0.00 ;0.01)	0.085
Glucose (mmol/L)	15	−0.002 (−0.01; 0.00)	0.472	0.001 (−0.01; 0.01)	0.490
	32	−0.003 (−0.01; 0. 00)	0.251	−0.001 (−0.01; 0.01)	0.751
	At birth	−0.005 (−0.01; 0.00)	0.081	−0.002 (−0.01; 0.01)	0.601
Insulin (pmol/L)	15	0.22 (0.03; 0.40)	**0.021**	0.25 (0.03; 0.46)	**0.026**
	32	0.15 (−0.02; 0.31)	0.076	0.11 (−0.08; 0.29)	0.249
	At birth	0.22 (0.05; 0.40)	**0.015**	0.23 (0.02; 0.44)	**0.035**
C-peptide (pmol/L)	15	0.99 (0.16; 1.82)	**0.020**	0.71 (−0.27; 1.69)	0.154
	32	0.71 (−0.03; 1.44)	0.060	0.37 (−0.45; 1.20)	0.370
	At birth	0.55 (−0.29; 1.39)	0.198	0.24 (−0.72; 1.20)	0.620
Triglycerides (mmol/L)	15	0.00 (−0.00; 0.00)	0.925	0.00 (−0.00; 0.00)	0.957
	32	−0.00 (−0.00; 0.00)	0.743	−0.00 (−0.00; 0.00)	0.805
	At birth	−0.00 (−0.00; 0.00)	0.797	−0.00 (−0.00; 0.00)	0.945

Associations between visceral adipose tissue (cm^3^) at different time-points during pregnancy and offspring outcomes are presented as the change in outcome with 95% confidence intervals for a 1 cm^3^ increase in visceral adipose tissue. Model 1 is unadjusted; Model 2 is adjusted for maternal pre-pregnancy BMI, parity, and gestational weight gain at the specific time, gestational age, and randomization group. Significant values are bold.

The women were categorized into groups based on whether their VAT volume increased or decreased from GW 15 to the time of birth, with 33% of the women experiencing an increase and 66% experiencing a decrease. No differences between the groups were found for maternal age, gestational age, pre-pregnancy BMI, or total GWG. However, women who experienced a reduction in VAT delivered offspring with birthweights 161 grams higher than those whose VAT volume increased during pregnancy; these women also had significantly more VAT at GW 15. No significant differences in offspring BMI z-score and cord blood biomarkers were observed between the two groups (Table 3).

**Table 3:** Maternal and offspring characteristics in women who experienced an increase or a decrease in visceral adipose tissue (VAT) volume during pregnancy.

Maternal characteristics	*N*	Increasers	*N*	Decreasers	Difference	*P* value
VAT in GW 15 (cm^3^)	39	115.6 (45.1)	80	134.8 (45.1)	−19.2 (−36.65; −1.75)	**0.031**
SAT in GW 15 (cm^3^)	39	391.9 (125.0)	80	420.3 (120.0)	−28.4 (75.4; 18.7)	0.235
Change in VAT (cm^3^)	39	28.1 (32.6)	80	−33.6 (29.7)	61.7 (49.84; 73.54)	**<0.001**
Change in SAT (cm^3^)	39	−1.8 (79.0)	80	−45.0 (67.7)	43.2 (15.56; 70.93)	**0.002**
Maternal age (years)	39	30.5 (4.4)	80	31.0 (4.5)	−0.5 (−2.23; 1.23)	0.568
Gestational age	39	280.1 (10.1)	80	282.2 (10.1)	−2.03 (−5.95; 1.88)	0.306
Pre-pregnancy BMI (kg/m^2^)	39	33.4 (4.2)	80	34.1 (3.9)	−0.7 (−2.23; 0.88)	0.391
Total GWG (kg)	39	7.97 (6.45)	80	6.48 (4.99)	1.48 (−0.65; 3.62)	0.170
Change in glucose (mmol/L)	37	−0.16 (0.39)	75	−0.18 (0.34)	0.02 (−0.12; 0.17)	0.731
Change in triglycerides (mmol/L)	37	1.39 (0.86)	77	1.16 (0.64)	0.22 (−0.06; 0.51)	0.123
**Offspring outcome**						
Birthweight (g)	39	3509 ± 59.6	79	3669 ± 41.4	−161 (−307; −15)	**0.031**
BMI z-score	39	−0.22 ± 0.13	79	0.00 ± 0.09	−0.23 (−0.54; 0.09)	0.153
Glucose (mmol/L)	28	5.76 ± 0.26	49	5.28 ± 0.20	0.48 (−0.19; 1.15)	0.158
Insulin (pmol/L)	27	46.3 ± 7.68	49	49.6 ± 5.77	−3.27 (−22.6; 16.1)	0.736
C-peptide (pmol/L)	28	334 ± 33	49	371 ± 26	−37 (−122; 48)	0.392
Triglycerides (mmol/L)	28	0.55 ± 0.05	50	0.53 ± 0.04	0.02 (−0.12; 0.12)	0.781

Maternal characteristics for women who experienced an increase or decrease in visceral adipose tissue are presented as mean (SD) with a mean difference between the groups calculated as increasers minus decreasers with 95% confidence intervals. The average of offspring outcomes in the two groups are presented as estimated means ± SE. The difference between groups is presented as estimated mean differences between women who increased or decreased their visceral adipose tissue. Offspring outcomes were adjusted for total GWG, gestational age, randomization group, parity, and pre-pregnancy BMI. Significant values are bold.

*GWG* gestational weight gain, *SAT* subcutaneous adipose tissue, *VAT* visceral adipose tissue.

## Discussion

Maternal VAT was measured by MRI in GW 15, 32, and at birth, with no significant associations observed with offspring birthweight. However, women who experienced a decrease in VAT volume during pregnancy (who also entered pregnancy with a higher VAT volume) gave birth to offspring weighing 161 grams more than those who experienced an increase in VAT during pregnancy. We also observed a positive association between maternal VAT and offspring BMI z-score and insulin levels.

Our findings are conflicting with results from other studies showing a positive association between maternal VAT measured by ultrasound in the first (GW 12) or early second trimester (GW 16–19) with offspring birthweight [10, 12] and the risk of giving birth to an infant large for gestational age independent of early-pregnancy BMI [12]. Not only did we not confirm these associations in our study, but instead we found evidence that women with overweight or obesity who increased their VAT volume gave birth to infants with lower birthweight. In non-pregnant individuals, VAT accumulation is thought to occur when adipocytes in the SAT are unable to store excess calories as triglycerides [17, 18].

In non-pregnant individuals, Moreover, VAT has higher lipolysis rates than SAT in non-pregnant individuals [17]. A study with tissue biopsies in healthy pregnant women revealed that SAT has higher lipolysis rates than VAT; however, this difference did not persist after adjusting for cell size [19]. Due to the much higher amount of SAT, most fatty acids in the systemic circulation in humans are primarily derived from SAT [20]. It is reasonable to assume the same is true among pregnant individuals. Accordingly, it is reasonable to assume that if more of these fatty acids are “trapped” in maternal VAT, fewer will be available for the fetus, thereby limiting fetal growth. Therefore, VAT accumulation during pregnancy may serve as a metabolic “sink” of fatty acids, limiting their availability for the fetus.

The women in our study were healthy women with overweight or obesity, with no other diseases, and were guided to consume a healthy diet during pregnancy [14]. Both groups of women—those who increased and those who decreased their VAT volume—had similar GWG and within the Institute of Medicine recommendations of <9 kg [21]. The decrease in VAT and SAT during pregnancy is likely the result of increased lipolysis and release of fatty acids to supply the growing fetus. Interestingly, we observed a positive association between maternal VAT in GW 15 and at birth and offspring insulin levels. Similarly, maternal VAT in GW 15 was positively associated with offspring insulin and C-peptide levels at birth (model 1). C-peptide is an indicator of insulin production since it is secreted in equimolar amounts by the pancreas [22]. Increased maternal VAT early in and late pregnancy might be related to greater insulin production in the offspring. However, after adjusting for confounding variables, the associations between maternal VAT in GW 15 and offspring C-peptide levels were no longer significant. Neither was maternal VAT during pregnancy associated with other offspring blood biomarkers in the offspring at birth. Previous studies have shown a positive association between maternal BMI and maternal glucose homeostasis with offspring cord blood insulin and C-peptide levels in normoglycemic pregnancies [23–25], which supports the Pedersen hypothesis of higher maternal glucose and offspring growth [26]. However, it is currently unclear whether the mother directly influences offspring insulin sensitivity or whether offspring insulin sensitivity is a response to the available nutrients from the maternal diet. A recent study involving a small sample size of 23 offspring found a positive association between the thickness of maternal VAT during the first trimester, measured by ultrasound, and the levels of non-esterified fatty acids and triglycerides in cord blood. Conversely, there was a negative correlation with the HOMA score (a marker of insulin resistance), while no significant association was found with offspring glucose or insulin levels [10]. The inverse relationship between maternal VAT and the HOMA score could indicate that offspring from mothers with elevated VAT may be more sensitive to insulin and potentially promote growth. However, the study’s conclusions are tentative due to the small sample size, which limits the ability to control for additional confounders.

Our study had several strengths, including the serial assessments of maternal abdominal adiposity distribution during pregnancy performed by MRI, in tandem with measurements of maternal biomarkers and offspring weight and cord blood samples. Monitoring changes in VAT during pregnancy could provide insights into fetal growth patterns and potential health risks for the mother and offspring that go beyond those gained using metrics of basic maternal anthropometry. However, several limitations can impact the validity of our findings. A total of 208 women completed the APPROACH dietary intervention study and delivered a live infant. Of them, 119 completed all MRI scans, resulting in lower statistical power. This study is a secondary analysis of a dietary RCT, which may be a significant modifier of maternal VAT and metabolic endpoints. Although RCT group allocation was included as a covariate in the model, its influence may be multifaceted and not fully accounted for. Also, the specificity in the inclusion criteria of the participants may influence the extent to which our results can be applied to a broader population. Moreover, the physiological changes concomitant to advancing pregnancy, such as the rearrangement of abdominal compartments due to the growing fetus and uterus, can affect the consistency and reliability of the serial MRI scans.

In conclusion, this is the first study evaluating the relationship between offspring birthweight and metabolic biomarkers with maternal VAT measured by MRI at several time-points during pregnancy. The positive association between maternal VAT and offspring insulin levels in GW 15 and at birth, and the impact of the changes in VAT during pregnancy on birthweight, underscores the complex interplay between maternal regional fat distribution and fetal development.

## Supplementary information

Supplementary material

## Data Availability

The datasets generated and analyzed during the current study are not publicly available but will be made available from the corresponding author upon reasonable request.
