# Supplementary material for: Visceral adipose tissue during pregnancy in women with overweight or obesity and offspring metabolic health
Source: Int J Obes (Lond). 2025 Aug 25;49(11):2249–53. doi: 10.1038/s41366-025-01872-9 (PMC12583199; doi:10.1038/s41366-025-01872-9)
Supplement: Supplementary file 1 — Supplementary material [file 41366_2025_1872_MOESM1_ESM.docx]

|  | N | Mean (SD) |
| --- | --- | --- |
| **Energy intake, kJ/day** |  |  |
| GW 21^1^ | 117 | 7704 (1925) |
| GW 32^1^ | 116 | 7460 (1906) |
| **Fat, E%** |  |  |
| GW 21^1^ | 117 | 31.7 (7.3) |
| GW 32^1^ | 116 | 31.7 (7.7) |
| **Carbohydrates, E%** |  |  |
| GW 21^1^ | 117 | 46.5 (9.5) |
| GW 32^1^ | 116 | 46.3 (9.3) |
| **Protein, E%** |  |  |
| GW 21^1^ | 117 | 21.8 (6.3) |
| GW 32^1^ | 116 | 21.9 (6.6) |
| **Glycemic index** |  |  |
| GW 15^2^ | 119 | 53.4 (5.2) |
| GW 28^2^ | 118 | 48.4 (6.5) |
| GW 36^2^ | 116 | 49.6 (6.1) |

**Table S1: Maternal diet during pregnancy**

E%, percentage of total energy intake. ^1^Data from 24-h recall. ^2^Data from food frequency questionnaire.
